# Retinoic acid regulates the ubiquitin–proteasome system in a middle cerebral artery occlusion animal model

**DOI:** 10.1186/s42826-022-00123-6

**Published:** 2022-05-13

**Authors:** Ju-Bin Kang, Murad-Ali Shah, Dong-Ju Park, Phil-Ok Koh

**Affiliations:** Department of Anatomy, College of Veterinary Medicine, Research Institute of Life Science, Gyeongsang National University, 501 Jinju-daero, Jinju, 52828 South Korea

**Keywords:** Cerebral ischemia, Neuroprotection, Retinoic acid, Ubiquitin–proteasome system

## Abstract

**Background:**

Retinoic acid is a major metabolite of vitamin A and exerts beneficial effects including anti-oxidant and anti-inflammatory activities in neurons. The ubiquitin–proteasome system is an important biological system that regulates cell survival. Ubiquitination regulates protein degradation and plays an important role in oxidative stress. Deubiquitinating enzymes cleave ubiquitin from proteins and control ubiquitination-induced degradation. We detected decreases in ubiquitin carboxy-terminal hydrolase L1, ubiquitin thioesterase OTUB1, and proteasome subunit alpha types 1 and 3 in cerebral ischemic damage. In this study, we investigated whether retinoic acid regulates the expression of deubiquitinating enzymes ubiquitin carboxy-terminal hydrolase L1, ubiquitin thioesterase OTUB1, and proteasome subunit alpha types 1 and 3 in cerebral ischemic injury. Right middle cerebral artery occlusion (MCAO) was performed to induce cerebral ischemic damage in male rats. Retinoic acid (5 mg/kg) or vehicle was intraperitoneally injected every day from 4 days before surgery. Neurological behavioral tests were performed 24 h after MCAO, and right cerebral cortical tissues were collected.

**Results:**

MCAO damage caused neurological behavioral dysfunction, and retinoic acid alleviated these deficits. The identified proteins decreased in MCAO animals with vehicle, while retinoic acid treatment attenuated these decreases. The results of proteomic study were confirmed by a reverse transcription-PCR technique. Expressions of ubiquitin carboxy-terminal hydrolase L1, ubiquitin thioesterase OTUB1, and proteasome subunit alpha types 1 and 3 were decreased in MCAO animals treated with vehicle. Retinoic acid treatment alleviated these MCAO-induced reductions. The ubiquitin–proteasome system plays an essential role in maintaining cell function and preserving cell shape against ischemic damage.

**Conclusions:**

These findings suggest that retinoic acid regulates ubiquitin- and proteasome-related proteins including ubiquitin carboxy-terminal hydrolase L1, ubiquitin thioesterase OTUB1, and proteasome subunit alpha types 1 and 3 in a brain ischemia model. Changes in these proteins are involved in the neuroprotective effects of retinoic acid.

## Background

Retinoic acid is a metabolite of vitamin A that mediates cell development and cell growth [1, 2]. It plays important roles including anti-inflammation, anti-apoptosis, and anti-oxidant [3, 4]. In the central nervous system, retinoic acid is involved in axonal growth and neuronal differentiation [5, 6]. It exerts neuroprotective effects by modulating neurodegeneration and neuroinflammation [7, 8]. Retinoic acid attenuates blood brain barrier disruption caused by ischemic damage because it has the advantage of easily passing through the blood brain barrier [9]. Furthermore, it improves tissue plasminogen activator-induced intracerebral hemorrhage and neurological deficits [9]. We recently reported that retinoic acid improves neurological deficits and infarction and has an anti-apoptotic function against focal cerebral ischemia [10]. Moreover, retinoic acid has neuroprotective effects in neurodegenerative diseases such as stroke, Alzheimer’s disease, and Parkinson’s disease [7, 11, 12].

The ubiquitin–proteasome system is catalytic machinery that degrades numerous cellular proteins. It is an essential system that acts extensively to regulate basic cell processes and cell survival. Proteasomes are protein complexes that break down damaged proteins by proteolysis. Misfolded or damaged proteins were degraded by tagging them with a small protein called ubiquitin. Ubiquitination and proteosomal degradation are important in the response to oxidative stress. Oxidative stress prevents proteasome activity and induces cell degradation and apoptosis. Damaged protein accumulation contributes to the pathogenesis in inflammatory responses, neurodegenerative diseases, and cardiovascular diseases [13, 14]. Ubiquitination also regulates membrane trafficking along with protein degradation and is counterbalanced by deubiquitinating enzymes [15, 16]. Deubiquitinating enzymes are proteases that reversely modify proteins by reducing ubiquitin or ubiquitin-like molecules [17]. Thus, they are considered regulators of ubiquitination-mediated degradation [16]. We identified decrease of deubiquitinating enzymes ubiquitin carboxy-terminal hydrolase L1 and ubiquitin thioesterase OTUB1 in cerebral ischemic damage caused by middle cerebral artery occlusion (MCAO). Decrease of proteasome subunit alpha types 1 and 3 was confirmed in stroke animal models. We elucidated the neuroprotective effect of retinoic acid by preventing apoptotic cell death [10]. Furthermore, it has been reported that retinoic acid regulates the ubiquitin–proteasome system [18]. Although the neuroprotective effect of retinoic acid has been demonstrated, its neuroprotective mechanisms are complex and vague. We propose that retinoic acid regulates ubiquitin–proteasome associated proteins and contributes to neuroprotection in ischemic brain damage. Thus, the aim of this study was to investigate whether retinoic acid regulates ubiquitin- and proteasome-related proteins ubiquitin carboxy-terminal hydrolase L1, ubiquitin thioesterase OTUB1, and proteasome subunit alpha types 1 and 3 in an animal model of stroke.

## Results

### Alleviation of neurological behavioral disorders by retinoic acid in MCAO animal model

**Fig. 1:**
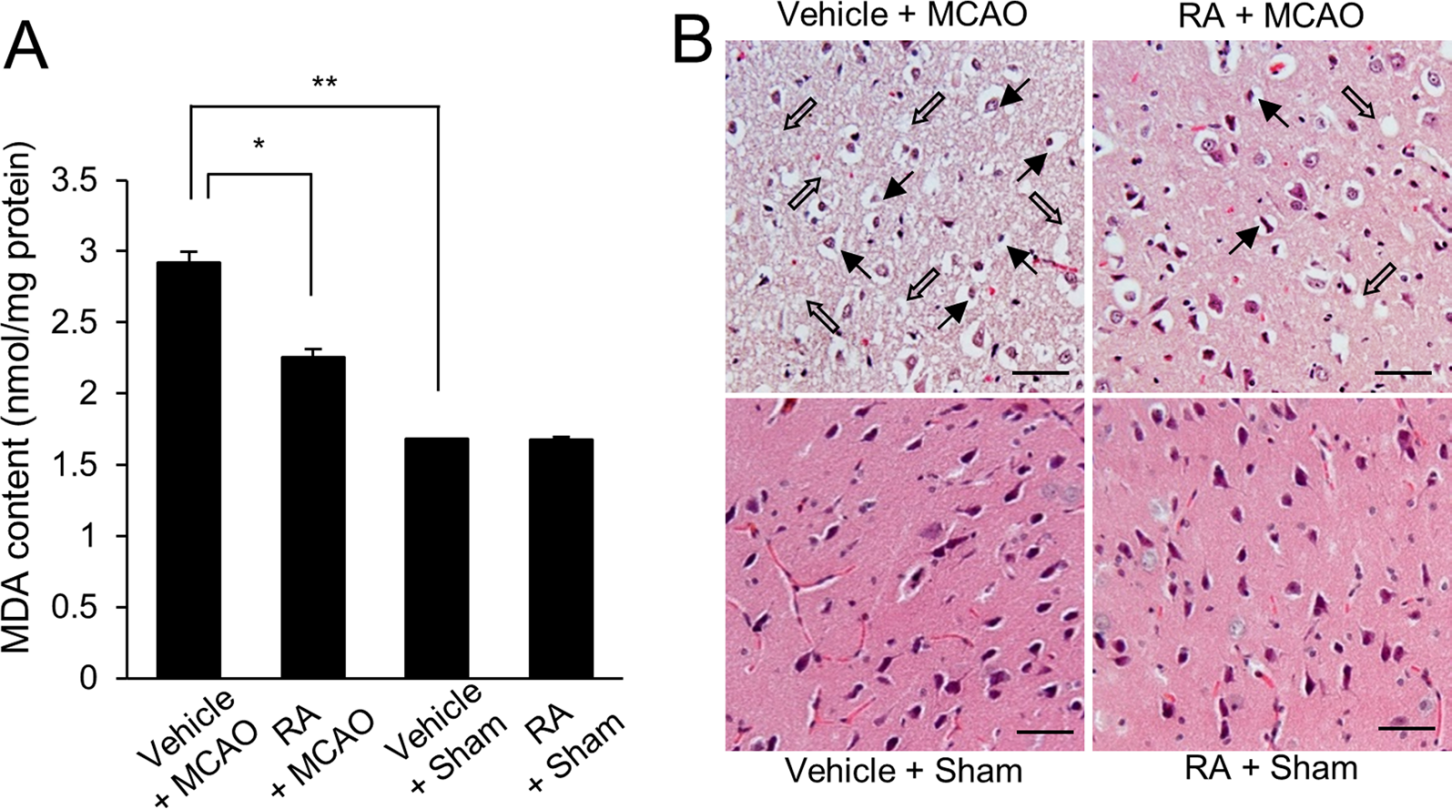
Malondialdehyde (MDA) analysis (**A**) and hematoxylin and eosin staining (**B**) in the right cerebral cortex of from vehicle + middle cerebral artery occlusion (MCAO), retinoic acid (RA) + MCAO, vehicle + sham, and RA + sham animals. RA alleviated the increase in MDA levels and histopathological changes caused by MCAO damage. The filled arrows indicate condensed nuclei and open arrows indicate vacuolated and swollen cytoplasm (B). Data (*n* = 5) are presented as mean ± standard error of the mean (S.E.M). *p < 0.05, **p < 0.01

We performed neurological behavioral tests to identify the neuroprotective function of retinoic acid during MCAO damage. We performed a variety of neurological behavioral tests, including neurological deficit scoring test, the corner test, and the grip strength test. We confirmed that MCAO damage causes neurological behavioral disorders, and retinoic acid treatment improves these damages. Specifically, MCAO damage induced neurological behavior deficits such as movement disorder, circling to contralateral side, and seizures. Neurological deficit scores were 3.27 ± 0.182 and 1.80 ± 0.175 in vehicle + MCAO animals and retinoic acid + MCAO animals. There was no significant difference in neurological deficit scores between vehicle + sham and retinoic acid + sham animals. The corner test results showed a preference for the direction of turn by right or left stimuli. The number of right turns was 9.20 ± 0.200 in vehicle + MCAO and 6.60 ± 0.214 in retinoic acid + MCAO animals. The number of left turns was almost the same in vehicles + MCAO and retinoic acid + MCAO animals. The response of the right or left stimuli is almost identical in sham-operated animals. MCAO surgery was performed in the right brain and the grip strength of the left forelimb was significantly reduced. This reduction was mitigated in retinoic acid-treated animals. The grip strength of the left forelimb was 0.13 ± 0.012 kg in vehicle + MCAO animals and 0.28 ± 0.015 kg in retinoic acid + MCAO animals. Overall, Table 1 describes the results of the neurological deficit scoring test, the corner test, and the grip strength test. Malondialdehyde (MDA) assay was performed to show the change of oxidative stress between vehicle + MCAO animals and retinoic acid + MCAO animals. MDA level was increased in vehicle + MCAO animals, retinoic acid treatment alleviated this increase. MDA levels were 2.92 ± 0.08 in vehicle + MCAO animals and 2.26 ± 0.03 in retinoic acid + MCAO animals (Fig. 1A). These results demonstrated that retinoic acid has anti-oxidative effect in cerebral ischemia. The results of hematoxylin and eosin staining showed histopathological lesions of the cerebral cortex due to MCAO damage (Fig. 1B). We observed normal neurons with large round nuclei, well-developed dendrites, and pyramidal shape in cerebral cortex of sham-operated animals regardless of vehicle or retinoic acid treatment. However, we observed the serious histopathological changes in shrunken dendrites, condensed nuclei, and cytoplasmic vacuole in vehicle-treated animals with MCAO damage. These changes were attenuated by retinoic acid.

**Table 1:** Results of neurological behavioral tests in middle cerebral artery occlusion

Test	Experimental group			
	Vehicle + MCAO	Retinoic acid + MCAO	Vehicle + Sham	Retinoic acid + Sham
Neurological deficit scoring test	3.27 ± 0.182**	1.80 ± 0.175**	0.00 ± 0.000	0.00 ± 0.000
Corner test (numbers to right turn)	9.20 ± 0.200**	6.60 ± 0.214**	4.80 ± 0.175	4.87 ± 0.192
Corner test (numbers to left turn)	0.80 ± 0.200**	3.40 ± 0.214**	5.20 ± 0.175	5.13 ± 0.192
Grip strength test (kg, right)	0.60 ± 0.014**	0.60 ± 0.015*	0.60 ± 0.013	0.61 ± 0.013
Grip strength test (kg, left)	0.13 ± 0.012**	0.28 ± 0.015*	0.59 ± 0.013	0.60 ± 0.013

Data (*n* = 10) are represented as the mean ± S.E.M. * p < 0.05, ** p < 0.001 vs. vehicle + MCAO animals

### Identification of differentially expressed proteins by retinoic acid in MCAO animal model

**Fig. 2:**
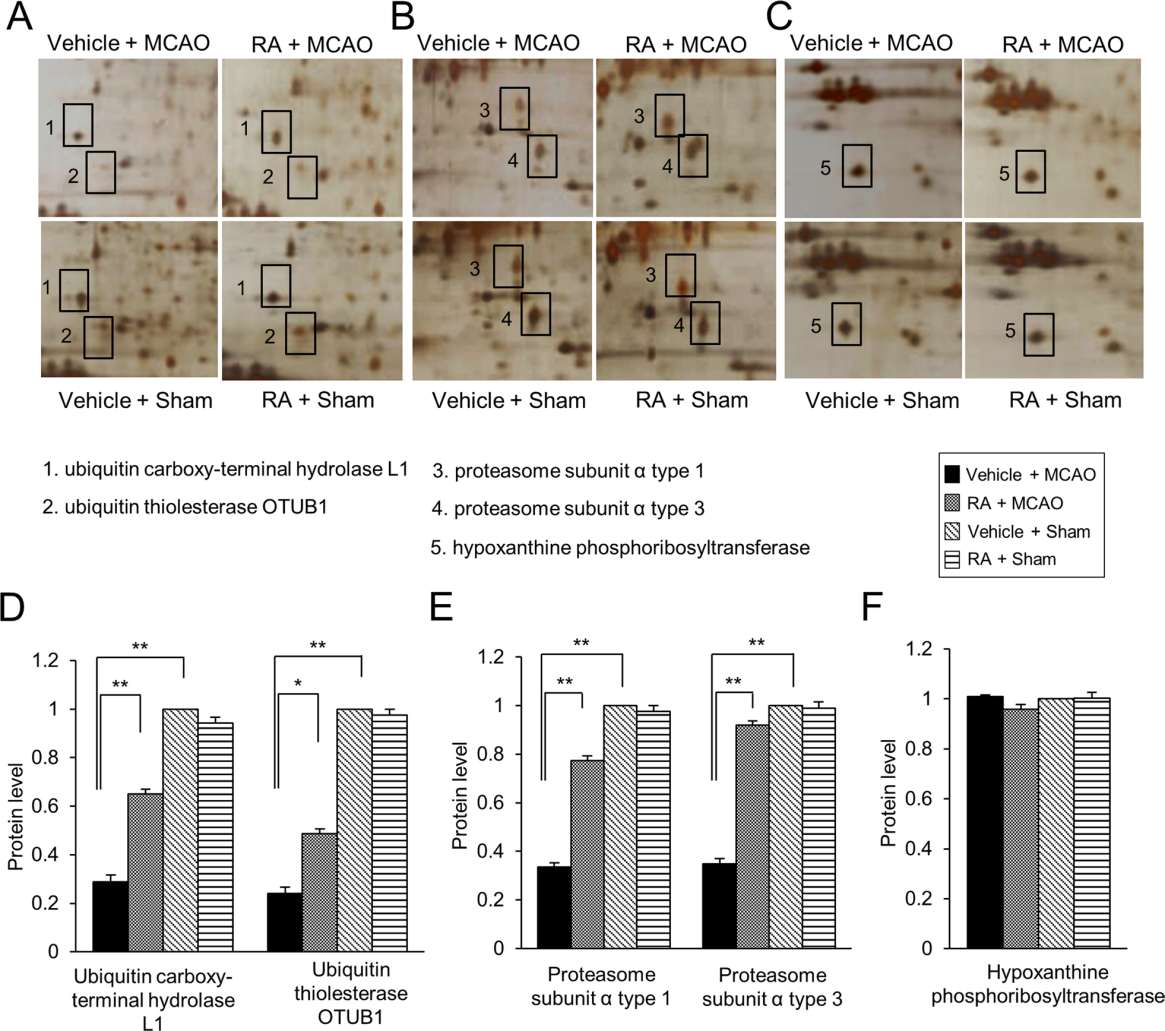
Identification of differentially expressed proteins by retinoic acid in middle cerebral artery occlusion animal model. Images of protein spots of ubiquitin carboxy-terminal hydrolase L1, ubiquitin thioesterase OTUB1 (**A**), proteasome subunit alpha types 1, proteasome subunit alpha types 3 (**B**), and hypoxanthine phosphoribosyltransferase (**C**) in the cerebral cortex from vehicle + middle cerebral artery occlusion (MCAO), retinoic acid (RA) + MCAO, vehicle + sham, and RA + sham animals. Each square indicates protein spots. Intensities of protein spots (**D–F**) were analyzed with Image J software and normalized as a ratio of intensity of each group to that of vehicle + sham group. Data (*n* = 5 per group) are represented as the mean ± S.E.M. *p < 0.05, **p < 0.01

We observed change of ubiquitin carboxy-terminal hydrolase L1, ubiquitin thioesterase OTUB1, and proteasome subunit alpha type 1 and 3 expression between vehicle- and retinoic acid-treated animals with MCAO damage using a proteomic approach (Fig. 2A and B). We identified the hypoxanthine phosphoribosyltransferase protein as the internal control for staining. We found that there was no significant change in the intensity of these proteins among experimental aniamls. Table 2 represents isoelectric point and sequence coverage in identified proteins. MCAO damage decreased the above mentioned proteins, while retinoic acid treatment attenuated these decreases. Expression of these proteins showed no significant difference between sham-operated animals regardless of vehicle or retinoic acid treatment. We evaluated the expression levels of these proteins as the intensity of protein spots. The intensity of each group was evaluated as the ratio of intensity of vehicle + sham group. Expression levels of ubiquitin carboxy-terminal hydrolase L1 were 0.29 ± 0.03 and 0.65 ± 0.02 in vehicle + MCAO and retinoic acid + MCAO animals, respectively (Fig. 2C). Ubiquitin thioesterase OTUB1 levels were 0.24 ± 0.03 in vehicle + MCAO animals and 0.49 ± 0.02 retinoic acid + MCAO animals (Fig. 2C). Moreover, proteasome subunit alpha type 1 levels were 0.34 ± 0.02 in vehicle + MCAO animals and 0.77 ± 0.02 retinoic acid + MCAO animals (Fig. 2D). Proteasome subunit alpha type 3 levels were 0.35 ± 0.02 and 0.92 ± 0.03 in vehicle + MCAO and retinoic acid + MCAO animals, respectively (Fig. 2D).

**Table 2:** Identified of ubiquitin–proteasome system related proteins in cerebral cortex of middle cerebral artery occlusion

Spot no.	Protein name	Accession no	Mw (Da)	pI	Mass matched	Sequence coverage (%)
1	Ubiquitin carboxy-terminal hydrolase L1	Q7TQI3	31.27	4.85	11/66	58
2	Ubiquitin thiolesterase OTUB1	B2RYG6	31.27	4.8	14/39	61
3	Proteasome subunit alpha type 1	P18420	29.52	6.15	7/117	35
4	Proteasome subunit alpha type 3	P18422	28.40	5.3	7/112	27
5	Hypoxanthine–guanine phosphoribosyltransferase	P27605	20.00	4.6	6/110	43

Protein names and accession numbers are listed according to the SWISS-PROT database

MW, molecular weight; pI, isoelectric point

### Reverse transcription‑PCR analysis of identified proteins in retinoic acid treated animals with MCAO

**Fig. 3:**
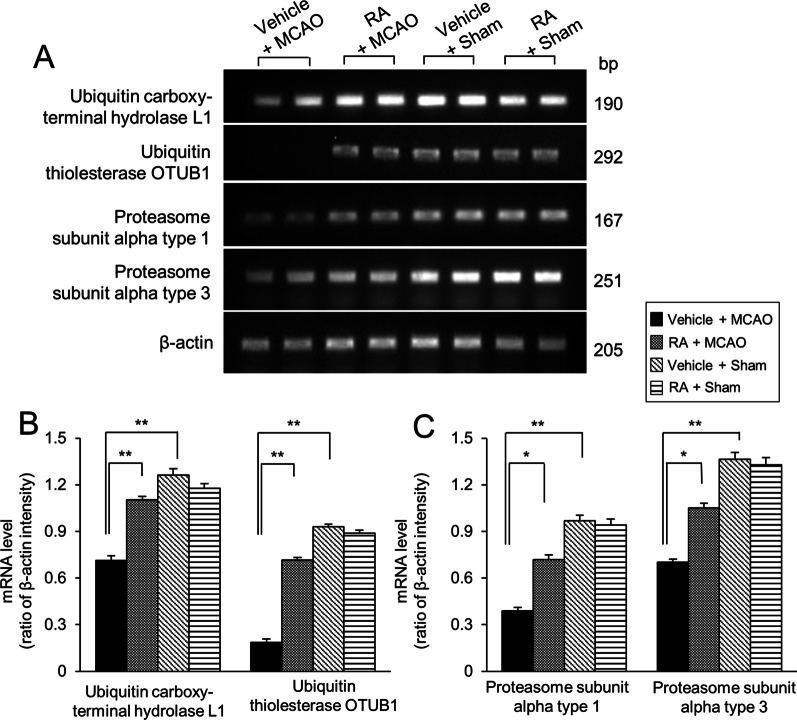
Reverse transcription‑PCR analysis of identified proteins in retinoic acid treated animals with middle cerebral artery occlusion animal model. Image of reverse transcription-PCR products (**A**) of ubiquitin carboxy-terminal hydrolase L1, ubiquitin thioesterase OTUB1, and proteasome subunit alpha types 1 and 3 in the cerebral cortex from vehicle + middle cerebral artery occlusion (MCAO), retinoic acid (RA) + MCAO, vehicle + sham, and RA + sham animals. The band intensity (**B** and **C**) of the reverse transcription-PCR product is expressed as a ratio of β-actin product intensity. Data (*n* = 5 per group) are represented as the mean ± S.E.M. *p < 0.05, **p < 0.01

Reverse transcription-PCR (RT-PCR) analysis showed changes in gene expression in MCAO-operated animals (Fig. 3A). MCAO damage reduced the expression of these genes compared to those in sham-operated animals, and retinoic acid treatment alleviated these decreases. However, no significant difference was observed in sham animals with vehicle or retinoic acid. We evaluated the expression levels of these genes using the intensity of the PCR products. Ubiquitin carboxy-terminal hydrolase L1 mRNA levels were 0.71 ± 0.03 in vehicle + MCAO animals and 1.10 ± 0.02 retinoic acid + MCAO animals (Fig. 3B). Ubiquitin thioesterase OTUB1 mRNA levels were 0.19 ± 0.02 and 0.72 ± 0.02 in vehicle + MCAO and retinoic acid + MCAO animals, respectively (Fig. 3B). Proteasome subunit alpha 1 mRNA levels were 0.39 ± 0.02 in vehicle + MCAO animals and 0.72 ± 0.03 retinoic acid + MCAO animals (Fig. 3C). Proteasome subunit alpha 3 mRNA levels were 0.70 ± 0.02 and 1.05 ± 0.03 in vehicle + MCAO and retinoic acid + MCAO animals, respectively (Fig. 3C).

## Discussion

MCAO surgery was performed to induce focal cerebral ischemia. Permanent MCAO is known to cause ischemic damage in the cerebral cortex as well as striatum. However, damage to the cerebral cortex was more pronounced than damage to the striatum [19]. The cerebral cortex is involved in sensory, motor, cognitive, learning and memory functions [20, 21]. The cortical damage caused by ischemic strokes causes very fatal dysfunctions because the cerebral cortex performs various functions in the brain. Therefore, this study focused on the cerebral cortex of MCAO animals. We confirmed that retinoic acid exerts neuroprotective functions by various neurological behavioral test including neurological deficit scoring test, the corner test, and the grip strength test. This study showed that retinoic acid regulates the ubiquitin–proteasome system in cerebral ischemic damage. Our proteomic approach identified the reduction of deubiquitinating enzymes and proteasome including ubiquitin carboxy-terminal hydrolase L1, ubiquitin thioesterase OTUB1, and proteasome subunit alpha types 1 and 3 in a rat model of MCAO. Retinoic acid mitigates ischemic damage-induced decreases in these proteins. We also found that the expression of hypoxanthine phosphoribosyltransferase is the same in all animals. Hypoxanthine phosphoribosyltransferase is used as a housekeeping gene in cerebral ischemia models [22, 23]. So, we thought that the results of our proteomic study were reliable. The ubiquitin system modulates protein activity and various cellular processes [24]. Ubiquitin carboxy-terminal hydrolase L1 is selectively expressed in the brain [25]. It regulates ubiquitination-induced damage such as aggregated or oxidized proteins [26]. It has neuroprotective effects on oxidative stress and axonal damage [26, 27]. The reduction of ubiquitin carboxy-terminal hydrolase L1 degrades synaptic function and reduces axonal transport, which increases neuronal death in oxygen- and glucose-deficient conditions [26, 27]. Ubiquitin carboxy-terminal hydrolase L1 also plays an important role in the ubiquitin proteasome pathway of neurons [28]. Cerebral ischemic damage increases reactive lipid formation, causes protein degradation and ubiquitin proteasome pathway dysfunction, and reduces activity of ubiquitin carboxy-terminal hydrolase L1 [28–30]. Retinoic acid alleviates promyelocytic leukemia protein-induced cell death by activation of the ubiquitin–proteasome pathway [31]. We demonstrated that MCAO reduces ubiquitin carboxy-terminal hydrolase L1 expression in the cerebral cortex, and retinoic acid attenuates this reduction in cerebral ischemic damage. Ubiquitin carboxy-terminal hydrolase L1 expression plays an important role for neuroprotective function. Therefore, our findings suggest that preservation of ubiquitin carboxy-terminal hydrolase L1 expression by retinoic acid is related to neuroprotective effects of retinoic acid in focal cerebral ischemia.

Ubiquitin thioesterase OTUB1 is a deubiquitinating enzyme that plays an important role in various signaling pathways [32]. It interacts with ubiquitin protease and deubiquitinates molecules including p53, Akt, and snail [33]. Ubiquitin thioesterase OTUB1 is expressed mainly in the Lewy bodies of brain and exerts an anti-apoptotic effect against intracerebral hemorrhage [34]. It is also a major regulator of astrocyte activation by regulating IFN-γ in astrocytes by inhibiting IFN-γ-induced chemokines and pro-inflammatory molecules to attenuate autoimmune encephalitis [35]. Ubiquitin thioesterase OTUB1 also regulates the accumulation and phosphorylation of tau protein [36]. Abnormal tau phosphorylation influences tau structure and distribution and causes neurodegenerative diseases including Alzheimer’s disease and ischemic stroke [37, 38]. Cerebral ischemic damage causes tau protein dysfunction and increases neuronal cell death [38]. Retinoic acid attenuates astrocytes and microglia activations, prevents beta-amyloid deposition, and alleviates neuronal degeneration by regulating tau phosphorylation in Alzheimer's disease model [39]. We confirmed that MCAO reduces OTUB1 expression through a proteomic approach and RT-PCR analyses. Decrease in ubiquitin thioesterase OTUB1 affects deubiquitination in various proteins. However, retinoic acid mitigated the decrease in ubiquitin thioesterase OTUB1 expression in cerebral ischemic damage. We showed the regulation of ubiquitin thioesterase OTUB1 expression under the presence of retinoic acid in MCAO damage. Maintenance of ubiquitin thioesterase OTUB1 expression is important for cell survival against brain damage. Further studies are needed to determine the relationship between retinoic acid and ubiquitin thioesterase OTUB1 in ischemic damage, but we suggest that retinoic acid regulates the expression of ubiquitin thioesterase OTUB1, and that maintenance of this protein contributes to the neuroprotective effect of retinoic acid in cerebral ischemic damage.

Proteasomes are involved in important mechanisms including cell cycle and cell growth regulation, gene transcription, signal transduction, and apoptosis [40]. Proteasomes are protein complexes that degrade damaged proteins or misfolded proteins by proteolysis and are pivotal components of ubiquitin–proteasome systems. They are multicatalytic protease complexes with 20S core structures that contribute to complete assembly of the 20S proteasome complex and consist of subunit alpha and beta types. We identified decrease in proteasome subunit alpha proteins in cerebral cortex damage caused by MCAO. These proteins are mediated in proteolytic processes of most intracellular proteins [41]. Reduction of proteasome activity and proteasome expression leads to cellular denaturation and dysfunction, causing neurodegeneration [42]. Brain ischemic damage exacerbates production of oxidized and misfolded proteins, accumulates ubiquitin-containing proteins, and consequently damages the protein degradation pathway. Ischemic condition leads to DNA damage by over-generating reactive oxygen species [43]. DNA damage reduces proteasome subunit alpha and controls proteolytic activity [44]. MCAO damage induces reduction of proteasome subunit alpha types 1 and 3, and retinoic acid alleviates decreases in these proteins. We previously showed decrease of proteasome subunit alpha in glutamate-exposed cerebral cortex [45]. Decrease of these proteins is involved in cerebral cortex damage during neuronal development [45]. Proteasome subunit alpha types 1 and 3 are subunits of the 20S proteasome involved in assembly of the 20S proteasome complex. Our finding shows that retinoic acid alleviates MCAO-induced reduction in proteasome subunit alpha types 1 and 3 in cerebral ischemia. This is the first report of change in proteasome subunit alpha types 1 and 3 in cerebral ischemic damage. Retinoic acid inhibits oxidative stress against cerebral ischemia and protects neuron from damage. Ischemic damage accumulates oxidized proteins and decreases proteasome activity [46]. Thus, the results of this study demonstrate that alleviation of proteasome subunit alpha types 1 and 3 reduction by retinoic acid maintains proteasome activity and protects neurons from oxidative stress in brain ischemic damage. In this study, our findings showed that retinoic acid modulates the expression levels of ubiquitin carboxy-terminal hydrolase L1, ubiquitin thioesterase OTUB1, and proteasome subunit alpha types 1 and 3 in MCAO-induced ischemic brain injury. Although further studies are needed to determine the biochemical relationship between retinoic acid and ubiquitin–proteasome system, our results demonstrated the regulation of these proteins by retinoic acid in cerebral cortical damage caused by MCAO. Therefore, these results suggest that retinoic acid regulates the ubiquitin–proteasome system and contributes to neuronal recovery and neuroprotection in ischemia.

## Conclusions

This study showed that retinoic acid alleviates neurological behavioral disorders caused by MCAO damage. Retinoic acid prevents the reduction of ubiquitin carboxy-terminal hydrolase L1, ubiquitin thioesterase OTUB1 and, proteasome subunit alpha types 1 and 3 expression induced by MCAO damage. These proteins are associated with ubiquitin–proteasome systems, suggesting that retinoic acid can perform neuroprotective functions by regulating ubiquitin–proteasome systems in ischemic brain injury.

## Methods

### Experimental animals and drug treatment

Male Sprague-Dawley rats (200–220 g, *n* = 52) were obtained from Samtako Co. (Animal Breeding Center, Osan, Korea). All animals were bred for a week to adapt to new environments under controlled temperatures (25 °C), humidity (60–70%), and light (12 h/12 h light/dark cycle), and allowed free access to feed and water. Animals were randomly divided into four groups; vehicle + MCAO, retinoic acid + MCAO, vehicle + sham, and retinoic acid + sham group. Retinoic acid (5 mg/kg, Sigma-Aldrich, St. Louis, MO, USA) was dissolved in solvent solution (polyethylene glycol, 0.9% NaCl, and ethanol; each volume 70%/20%10%) and injected intraperitoneally for four consecutive days before MCAO surgery [9]. Vehicle treated groups were injected with a solvent solution without retinoic acid.

### MCAO surgery

MCAO surgery was performed to induce focal cerebral ischemia by following a previously described method [47]. Rats were anesthetized with Zoletil (50 mg/kg, Virbac, Carros, France) and placed on a surgical operating table. A median incision was performed on the neck of the animals, and the incision further extended with scissors to explore and isolate the right common carotid artery (CCA) from nearby muscles, tissues, and nerves. The external carotid artery (ECA) and the internal carotid artery (ICA) were also properly exposed and separated from muscles and tissues. The right CCA was occluded with a non-traumatic microvascular clamp and the proximal end of the ECA was cut. A 4-0 nylon suture with a rounded end was carefully inserted into the ECA. The nylon suture was inserted in the ICA until it was resisted. The middle cerebral artery was blocked by nylon. The inserted nylon suture and the cut ECA were sutured for fixation, and the cut skin was sutured with black silk. The animals were kept on heating pad to maintain the body temperature during and after the operation. The same surgical operation was performed in sham animals, except for nylon suture insertion. Animals were sacrificed 24 h after MCAO surgery and cerebral cortical tissues were collected for further studies.

### Neurological deficit scoring test

We performed a neurological deficit score test 24 h after MCAO surgery through the five-point system as a previously described manual [48]. Neurological deficit scores were given to the animals according to their neurological condition from 0 to 4 as given below: animals showing no neurological deficit and remained in normal position (0), animals showing very little neurological deficit like halfway extension of the contralateral forelimb (1), animals showing adequate neurological deficit like circling to the contralateral side (2), animals showing severe neurological deficit such as showing signs of seizures and falling to the contralateral side (3), and animals remaining unconscious and showing no sign of movement (4).

### Corner test

The corner test is used to evaluate posture and sensorimotor asymmetry in MCAO-induced brain damage [49]. The apparatus used for the corner test consists of two whiteboards (30 × 20 × 1 cm^3^) kept at an angle of 30° to each other. A little space is required at the corner to allow free movement of the rats during the test. The animals were removed one by one from their cages and placed between the two whiteboards. The animals were allowed to move freely into the corner and turned left or right to get back to the wider side of the whiteboards when their vibrissaes were touched. A total of 10 experiments were conducted on each animal and the number of left and right turns was recorded. Animals that would turn to the left and right side at the same ratio were approved for the corner test. All animals were trained for 7 days before MCAO surgery. After 24 h of MCAO surgery, the corner test was performed and the number of left and right turns were recorded for each animal.

### Grip strength test

We used a grip strength meter (Jeung Do Bio & Plant Co., Ltd., Seoul, Korea) for evaluation of the grip strength test in the left and right forelimbs [50]. The procedure was started by placing the left or right forelimb of rats on the metal mesh of the gripper and adjusting it zero. After adjusting, the animals’ tails were grabbed and the animals were pulled back. As a result, the animals gripped the mesh with their paws and held it tight with maximum force. The test was repeated five times and the maximum force tension of each animal was recorded.

### MDA measurement

MDA analysis was performed to investigate oxidative stress. MDA is the final product of lipid peroxidation. Lipid peroxidation (MDA) colormetric/fluorometric assay kit (BioVision Inc., Milpitas, CA, USA) was used for MDA assay. We conducted this experiment according to the manufacture’s instruction (BioVision Inc., Milpitas, CA, USA). Cerebral cortices (10 mg) were homogenized in MDA lysis buffer with butylated hydroxytoluene and were centrifuged at 13,000*g* for 10 min at 4 °C. Supernatants were collected and mixed with thiobarbituric acid with 30% glacial acetic acid. Mixtures were incubated for 1 h at 95 °C and cooled in ice for 10 min. MDA concentration was measured by spectrophotometer at absorbance of 532 nm.

### Hematoxylin and eosin staining

Brain tissues were fixed in 4% neutral buffered paraformaldehyde, washed with tap water for overnight, dehydrated with graded series of ethanol from 70 to 100%, and cleaned with xylene. They were infilterated with paraplast (Leica, Wetzlar, Germany) and embedded using paraffin embedding center (Leica). Paraffin blocks were cut into 4 µm thicknesses using rotary microtome (Leica) and paraffin ribbons were mounted on slide glass. Sections were dried on slide warmer (Thermo Fisher Scientific, Waltham, MA, USA). They were dipped in xylene to remove paraffin, hydrated with graded series of ethanol from 100 to 70%, and washed with tap water. Sections were stained with Harris’ hematoxylin solution (Sigma-Aldrich) for 5 min and washed with tap water. They were subsequently dipped in 1% hydrochloric acid with ethanol, washed with water, and dipped in 1% ammonia water. Sections were washed with water, stained with eosin Y solution (Sigma-Aldrich) for 2 min, and washed with water. They were dehydrated with graded series of ethanol from 70 to 100%, cleaned with xylene, and coverslipped with mounting solution (Thermo Fisher Scientific). The stained tissues were observed and photographed under an Olympus microscope (Olympus, Tokyo, Japan).

### 2-Dimensional gel electrophoresis

Right cerebral cortex tissues were isolated and immediately kept at -70 °C. They were homogenized in lysis buffer (8 M urea, 4% CHAPS, ampholytes, and 40 mM Tris–HCl) with 200 µM phenylmethylsulfonyl fluoride and were centrifuged at 15,000*g* for 15 min at 4 °C. The supernatants from the centrifugation were collected. The collected supernatants were precipitated with 10% trichloroacetic acid for 30 min at room temperature and were centrifuged at 14,000*g* for 15 min at 4 °C. The supernatants from the centrifugation were removed. The obtained pellets were washed with acetone and were dissolved in a sample buffer [8 M urea, 4% CHAPS, 0.2% amphoteric solution, 40 mM Tris–HCl, 2 μg/ml dithiothreitol (DTT)]. The protein concentration for each protein was analyzed with a Bradford assay (Bio-Rad, Hercules, CA, USA) by using bovine serum albumin as the standard. A total of 50 μg was loaded into an immobilized pH gradient (IPG) gel strip (pH 4–7, 17 cm, Bio-Rad) after mixing it with rehydration buffer (8 M urea, 2% CHAPS, 20 mM DTT, 0.5% IPG buffer, bromophenol blue) for 15 h at room temperature. First isoelectric focusing was performed using Ettan IPGphor 3 System (GE Healthcare, Little Chalfont, Buckinghamshire, UK) by the following conditions: 250 V for 15 min, 10,000 V for 3 h, and then 10,000 to 50,000 V. The electrophoresed IPG strips were treated with equilibration buffer (6 M urea, 30% glycerol, 2% sodium dodecyl sulfate, 50 mM Tris–HCl, and bromophenol blue) containing 1% DTT for 10 min and keep with equilibration buffer containing 2.5% iodoacetamide for 10 min. The IPG strips were loaded into the top of the gradient gels (7.5–17.5%) and covered with agarose gel with bromophenol blue dye. The strips were electrophoresed at 10 mA with Protein-II XI electrophoresis equipment (Bio-Rad) at 15 °C until the blue dye reached the bottom of the gel. The gels were carefully removed from the glass and were fixed in a fixing solution (12% acetic acid and 50% methanol) for 2 h. The gels were washed two times with 50% ethyl alcohol for 20 min and were reacted with a 0.2% sodium thiosulfate solution for 1 min. They were washed with distilled water three times for 1 min and were stained with a silver nitrate solution (0.2% silver nitrate and 0.0003% formaldehyde) for 20 min The stianed gels were washed two times with distilled water and were developed in a developing solution (0.2% sodium carbonate and 0.0002% formaldehyde) until all the protein spots were visible on the gels. Developing was stopped using 1% acetic acid solution and the images of the gels were taken with a scanner (Agfa ARCUS 1200TM, Agfa-Gevaert, Mortsel, Belgium). The images were saved and the changes in intensities among different proteins were analyzed with the PDQest 2-D analysis software (Bio-Rad). Matrix-assisted laser desorption ionization time-of-flight (MALDI-TOF) was performed to identify the differently expressed proteins. Protein spots were cut from the gel, desalted using 30 mM potassium hexacyanoferate with 100 mM sodium thiosulfate, and washed with 10% acetic acid in 50% methanol solution. They were incubated with 50 mM ammonium bicarbonate and acetonitrile and dried in a vacuum centrifuge. Dried samples were dissolved in a reduction solution (10 mM DTT in 0.1 M ammonium bicarbonate) for 45 min at 56 °C and immersed with 0.1 M ammonium bicarbonate and acetonitrile. They were dried in a vacuum centrifuge for 20 min and incubated with digestion solution (12.5 ng/ml trypsin, 0.1% octyl beta-D glucopyranoside in 50 mM ammonium bicarbonate) for 12 h at 37 °C. Proteins were dried in vacuum centrifuge and dissolved with 1% trifluoroacetic acid in 66% acetonitrile and matrix solution (16 mg/ml alpha-cyano-4-hydroxinic acid, 4 mg/ml nitrocellulose in acetone). They were placed on a MALDI-TOF plate and MALDI-TOF was performed by a Voyager System DE-STR MALDI-TOF mass spectrometer (Applied Biosystem, Forster City, CA). Analyzed proteins were then identified with MS-Fit and ProFound software and confirmed by SWISS-PROT and NCBI online databases. Intensities of protein spots were analyzed with Image J software (National Institutes of Health, Bethesda, MD, USA) and normalized as a ratio of intensity of each group to that of vehicle + sham group.

### Reverse transcription-polymerase chain reaction

Right cortical tissues were homogenized in Trizol reagent (Thermo Fisher Scientific), mixed with chloroform, and centrifuged at 13,000 g for 15 min at 4 °C. Supernants were collected and mixed with isopropanol. Mixtures were centrifuged at 13,000*g* for 20 min at 4 °C and supernatants were eliminated. Remained pellets were washed with 70% ethyl alcohol, and dissolved in RNase-free water. Total RNA (500 ng) was used for complementary DNA synthesis and reverse transcription step was performed using GoScript™ Reverse Transcriptase (Promega, Madison, WI, USA) according to the manual. RT-PCR was carried out as following protocol; denaturation for 5 min at 94 °C; 30 cycles of denaturation step at 94 °C for 30 s, annealing step at 54 °C for 30 s, elongation step at 72 °C for 1 min; and a final extension step for 5 min at 72 °C. Specific primers were used for amplification of the targeted genes (Table 3). The amplified genes were loaded into 1% agarose gel and electrophoresed for 15 min, electrophoresed agarose gel was visualized using Molecular Imager® Gel Doc XR System (Bio-Rad), and images were taken. The band intensities were analyzed with Image J software (National Institutes of Health, Bethesda, MD, USA) and normalized as a ratio of intensity of specific gene to that of β-actin.

**Table 3:** Sequence of the primers used for PCR amplification

Gene	Primer sequences (F, forward; R, reverse)	Product (bp)
Ubiquitin carboxy-terminal hydrolase L1	F: 5′-TGAAGCAGACCATCGGGAAC-3′ R: 5′-GAGTCATGGGCTGCCTGAAT-3′	190
Ubiquitin thiolesterase OTUB1	F: 5′-GCGACCACATCCACATCA-3′ R: 5′-ATGACCATTTACAACCACA-3′	292
Proteasome subunit alpha type 1	F: 5′-CCAACACAGCGATATGGCCG-3′ R: 5′-CTCTCCAGGTAAGTGCGAGC-3′	167
Proteasome subunit alpha type 3	F: 5′-GGCACTGGGTATGACCTGTC-3′ R: 5′-AAGGAACGAGCATCTGCCAA-3′	251
β-actin	F: 5′-TACAACCTTCTTGCAGCTCCTC-3′ R: 5′-CCTTCTGACCCATACCCACC-3′	205

### Statistical analysis

All experimental results are represented as means ± standard error of mean (S.E.M). Signal intensities were analyzed by SigmaPlot 4.0 (SPSS Inc., Point Richmond, CA, USA). The results of each group were compared by two-way analysis of variance (ANOVA) followed by post-hoc Scheffe’s test. Data was considered statistically significant when *p* value is less than 0.05.

## Data Availability

The data that support the findings of this study are available on request from the corresponding author on reasonable request.

## Abbreviations

CCA: Common carotid artery
DTT: Dithiothreitol
ECA: External carotid artery
ICA: Internal carotid artery
IEF: Isoelectric focusing
IPG: Immobilized pH gradient
MALDI-TOF: Matrix-assisted laser desorption ionization time-of-flight
MCAO: Middle cerebral artery occlusion
RT-PCR: Reverse transcription-polymerase chain reaction
